# Downregulation of host tryptophan–aspartate containing coat (TACO) gene restricts the entry and survival of *Leishmania donovani* in human macrophage model

**DOI:** 10.3389/fmicb.2015.00946

**Published:** 2015-10-13

**Authors:** Venkateswara Reddy Gogulamudi, Mohan Lal Dubey, Deepak Kaul, Venkata Subba Rao Atluri, Rakesh Sehgal

**Affiliations:** ^1^Department of Parasitology, Postgraduate Institute of Medical Education and Research, ChandigarhIndia; ^2^Department of Physiology, Tulane University School of Medicine, New Orleans, LAUSA; ^3^Department of Experimental Medicine and Biotechnology, Postgraduate Institute of Medical Education and Research, ChandigarhIndia; ^4^Department of Immunology, Herbert Wertheim College of Medicine, Florida International University, Miami, FLUSA

**Keywords:** leishmaniasis, TACO gene, Coronin-1A, host–parasite interactions, intracellular parasite, vitamin D, retinoic acid, chenodeoxycholic acid

## Abstract

*Leishmania* are obligate intracellular protozoan parasites of mammalian hosts. Promastigotes of *Leishmania* are internalized by macrophages and transformed into amastigotes in phagosomes, and replicate in phagolysosomes. Phagosomal maturation arrest is known to play a crucial role in the survival of pathogenic *Leishmania* within activated macrophages. Recently, tryptophan–aspartate containing coat (TACO) gene has been recognized as playing a central role in the survival of *Mycobacterium tuberculosis* within human macrophages by arresting the phagosome maturation process. We postulated that a similar association of TACO gene with phagosomes would prevent the vacuole from maturation in the case of *Leishmania*. In this study we attempted to define the effect of TACO gene downregulation on the entry/survival of *Leishmania donovani* intracellularly, by treatment with Vitamin D_3_ (Vit.D_3_)/Retinoic acid (RA) and chenodeoxycholic acid (CDCA)/RA combinations in human THP-1 macrophages (*in vitro*). Treatment with these molecules downregulated the TACO gene in macrophages, resulting in reduced parasite load and marked reduction of disease progression in *L. donovani* infected macrophages. Taken together, these results suggest that TACO gene downregulation may play a role in subverting macrophage machinery in establishing the *L. donovani* replicative niche inside the host. Our study is the first to highlight the important role of the TACO gene in *Leishmania* entry, survival and to identify TACO gene downregulation as potential drug target against leishmaniasis.

## Introduction

Leishmaniasis is a major public health issue in many parts of the world, affecting over 20 million people worldwide. About 350 million people are at risk of being infected with leishmaniasis, and 1.5-2 million children and adults develop the disease each year (Desjeux, 2004). This disease is caused by a parasite, which belongs to the genus *Leishmania*. All species of the genus *Leishmania* are obligate intracellular parasites that pass their life cycle in two hosts: the mammalian host, and the insect vector, the female sandfly. In human and other mammalian hosts, they multiply within macrophages, in which they occur exclusively in the amastigote form. Intracellular parasites have evolved through diverse mechanisms to enhance their survival and replication within host cells (Hackstadt, 2000). These mechanisms greatly involve adaptations for survival in different intracellular compartments that permit the parasites to avoid lysosomal killing. Although functions of most of these strategies remain unclear, the majority is expressed early on infectious process, suggesting that manipulation of the vacuole is critical to the outcome of the host-parasite interaction.

Tryptophan–aspartate containing coat (TACO) protein (a coat protein of phagosomes), also known as Coronin 1A, was shown to restrict the delivery of *Mycobacteria* to lysosomes (Ferrari et al., 1999). TACO/Coronin-1A belongs to the tryptophan–aspartate (WD) repeat containing family proteins (Neer et al., 1994; Suzuki et al., 1995), some of which are implicated in cytoskeletal organization, signal transduction, motility (Burrows et al., 1995), cytokinesis and vesicle formation (Pryer et al., 1993). Specifically, TACO is a 57 kDa polypeptide that binds to actin and is involved in cytoskeletal modulation (Gatfield et al., 2005), cytokinesis and intracellular membrane transport (Rybakin and Clemen, 2005). TACO is present on the cytoplasmic face of the plasma membrane and is retained by vacuoles carrying mycobacteria through phagocytosis, results in shielding of the mycobacteria within phagosomes of the host molecule by inhibiting its fusion with any other organelle, including lysosomes (Ferrari et al., 1999). Therefore, the active retention of TACO around the mycobacterial phagosome prevents the delivery of this machinery, and the pathogen continues to survive and replicate within the TACO armored phagosome. Several lines of evidence have been developed to show that TACO becomes activated in *Mycobacterium tuberculosis* infected cells, and this contributes to disease pathogenesis. Recent studies have shown that the downregulation of TACO leads to the suppression of mycobacteria infectivity and multiplication rate (Anand and Kaul, 2005).

However, the possible contribution of TACO in the inhibition of fusion of *Leishmania donovani*-containing phagosomes was not studied. Both *Mycobacterium* and *Leishmania* are intracellular organisms with macrophages as their primary target cells. These interference mechanisms are the main focus of this study. Dermine et al. (2000) suggested that it would be of interest to validate whether this TACO protein is also coupled with *Leishmania*-containing phagosomes. In addition to their limited availability, anti-leishmanial drugs have disadvantages such as toxicity, required prolonged treatment, challenge of drug resistance and high cost (Sundar et al., 2007). Attempts to develop newer synthetic compounds have met with a limited success. Thus, there is an urgent need to develop better anti-leishmanial drugs to supplement those in present use. Simple compounds like vitamins have not yet been explored as sources for possible anti-leishmanial agents. Vitamin D_3_ (Vit.D3), retinoic acid (RA), and chenodeoxycholic acid (CDCA) have been explored against *M. tuberculosis*; however, no scientific data is available on the utility of these endogenous molecules with regard to their anti-leishmanial activity *in vitro* or *in vivo*.

Taking all these findings into consideration, the aim of this study was to assess the effect of downregulation of TACO gene expression on entry and survival of *L. donovani* in human macrophages. The present study was designed to explore the inherent capacity of isoprenoid (CDCA, derived from mevalonate pathway) and vitamins to regulate TACO gene transcription, and their effect on entry and survival of *Leishmania* intracellularly. Based upon its previously described effects, this study has been designed to address these significant gaps in evidence that downregulation of TACO gene expression will lead to inhibition of *Leishmania* entry and survival in host macrophages. Such approach may help in the development of new, safe, effective, and inexpensive drug molecules, which can act at preventive and therapeutic levels against *Leishmania* infection.

## Materials and Methods

### Parasites

The standard strain of *L. donovani*: MHOM/IN/80/DD8 being maintained in the Department of Parasitology, PGIMER, Chandigarh, by serial *in vitro* culture and passaged through BALB/c mice for maintenance of virulence was used in the study.

### Macrophage Cell Line

THP-1, a human monocytic leukemia cell line was procured from NCCS, Pune, India. THP-1 monocytic cells were cultured in complete culture medium containing RPMI-1640 and HEPES (Invitrogen) supplemented with 10% fetal calf serum and 1% Penicillin (5000 U/ml)-streptomycin (5000 μg/ml; Sigma-Aldrich). The cells were kept in a humidified atmosphere at 37°C and 5% CO_2_. Cells were seeded in six-well plates (1 × 10^7^ cells/ml) in the complete medium. The cells were incubated with 30 nM/ml Phorbol 12-myristate 13-acetate (PMA, Sigma-Aldrich) for 72 h (Tsuchiya et al., 1982; Verstraelen et al., 2008), which induced differentiation of these cells into adherent macrophages. Plates were washed twice with pre-warmed RPMI-1640 to remove PMA, and 2000 μl of complete RPMI-1640 medium was then added. Differentiated cells were maintained until experiments were conducted.

### Conventional RT-PCR

After differentiation of THP-1 cells into macrophages as explained above, culture medium was aspirated and cells were rinsed with PBS. These cells were processed for total RNA extraction using TRI Reagent solution (Ambion, USA) based on acid guanidinium thiocyanate-phenol-chloroform method (Chomczynski and Sacchi, 1987). Quantity and quality of RNA were determined by reading absorbance at 260 and 280 nm. First-strand cDNA was synthesized from 1.5 μg of total cellular RNA using random hexamer primers and Moloney murine leukemia virus (M-MuLV) reverse transcriptase (RT) in a 20 μl reaction volume. Reagents were obtained from RevertAid^TM^ first strand cDNA synthesis kit (Fermentas, USA). 1 μl of cDNA was used as a template in each PCR reaction. cDNA of each sample was amplified using primers specific for TACO gene and carried out using glyceraldehyde phosphate dehydrogenase (GAPDH) as a control for RT efficiency. PCR reagents were obtained from MBI Fermentas and specific primers were obtained from MWG-Biotech. PCR was performed in a 25 μl reaction volume containing 1x PCR buffer, 2.5 mM MgCl_2_, 0.2 mM of each dNTP, 0.5 μM of each primer and 0.625 U of Taq (*Thermus aquaticus*) DNA polymerase. The primer sequences are described below. TACO (512 bp) Forward: 5′-CCAGTGCTATGGGATGTGCGCG-3′ Reverse: 5′-GACACGACTCGCTTGTCACGGC-3′ GAPDH (270 bp) Forward: 5′-AAGGCACAGACATGGTTGGT-3′ Reverse: 5′-TGGAAAGCAAACTGCCCTGA-3′.

### TACO Gene Sequencing

Polymerase chain reaction amplicons were detected by electrophoresis using 1.5% agarose gels and visualized using ethidium bromide. The amplified TACO gene PCR product of the expected size 512 bp was extracted from the agarose gel with a Mini Gel Extraction Kit (Qiagen). The PCR products were purified using Microspin columns (Amersham Pharmacia) prior to sequencing. Sequencing was done using specific primers and ABI PRISM Big Dye Terminator cycle sequencing ready reaction kit. The sequences were analyzed on an ABI PRISM 3730 DNA analyzer (Applied Biosystems, Foster City, CA, USA). The sequence of amplicon was confirmed by single direction using TACO primers.

### Determination of TACO mRNA Level

GAPDH gene was used as a control. The relative levels of TACO gene mRNA in Vit.D_3_/RA and CDCA/RA treated cells were determined by real-time PCR. The concentrations of molecules showing the maximum gene downregulation were used in further studies.

After treating the THP-1 cells with PMA as explained above, culture medium was aspirated and cells were rinsed with PBS. At the end of the incubation, the cells were incubated for 24 h in the presence of various stimuli: one set of cultures were pre-treated with Vit.D_3_/RA (Sigma–Aldrich, USA) at two doses (0.5/0.5, 1.0/1.0 μM), and another set were pre-treated with CDCA/RA in a dose dependent manner (25, 50, 75, and 100 μM of CDCA) in the constant presence of RA at 0.5 μM for 24 h at 37°C in 5% CO_2_. After 24 h of incubation at 37°C, real time PCR (relative quantification) was performed using LightCycler 480 SYBR Green I master mix (Roche) detection method to confirm the quantitative expression of TACO gene. GAPDH was used as internal control. Reaction mixture containing 1 μl (20-30 ng) of purified cDNA, 10 μl of 2X master mix (SYBR Green1 Dye), 10 picomoles of each primer of TACO and GAPDH genes was prepared and total volume was made up to 20 μl in nuclease free water. The cDNA synthesis reaction was performed with RNA along with all the other components but without RT enzyme. A “no-RT” control would yield a PCR product in case of genomic DNA contamination of the RNA sample. In order to nullify the variation in samples, we have presented the data of our quantitative analysis as ΔCp (ΔCp = Cp Target gene – Cp GAPDH). Cp (crossing point) value is inversely proportional to the gene level, thus Cp has an indirect relationship with the relative levels of the target gene; the greater the value, the lower the expression level.

Relative gene expression of TACO mRNA was performed by real-time PCR using SYBR Green-I master detection method using specific primers. To minimize the loading variations, GAPDH, an internal control gene, was run in all the samples from different groups. The real-time PCR analysis of transcripts for GAPDH in the Vit.D_3_/RA and CDCA/RA in different doses showed similar amplification and dissociation curves. In order to nullify the due variation in samples, the data was normalized to GAPDH, which served as internal control (ΔCp = Cp Target gene – Cp GAPDH), then again normalized to control (ΔΔCp = ΔCp Test sample – ΔCp Control sample), and final data was presented in the form of relative fold change by 2^-ΔΔCp^.

### Western Blot Analysis

After differentiation of THP-1 cells into macrophages as explained above, culture medium was aspirated and cells were rinsed with PBS. Adhered macrophages were detached by adding 1 ml acutase (PAA Laboratories, UK) at 37°C for 10 min. The cells were pelleted and the supernatant was discarded. The cells were washed gently by resuspending in 1 ml of PBS, and pelleted at low speed. Cell pellet was lysed in 1 ml of lysis buffer [50 mM Tris-HCl, pH 7.6, 150 mM NaCl, 5 mM EDTA, 1 mM ethylene glycol-bis (β-aminoethyl ether) *N*, *N*, *N*′, *N*′-tetraacetic acid (EGTA), 0.1% Triton X-100, 0.3% w/v β-mercaptoethanol, 0.28 TIU/ml aprotinin, 2 mM phenylmethylsulphonyl fluoride (PMSF), leupeptin 0.5 mg/ml and containing 100 μl protease inhibitor cocktail (Roche, Mannheim, Germany)] for 3 min at 4°C. Western blotting was carried out using a Mini Trans-blot electrophoretic transfer assembly (Bio-Rad Laboratories, USA; Towbin et al., 1979). After the transfer, PVDF membrane was blocked with 5% PBS-skimmed milk for 2 h and incubated with primary antibody (Novus Biologicals, USA) diluted in 5% PBS-skimmed milk overnight at 4°C. The membrane was then washed twice with PBST, once with PBS and incubated with Horseradish peroxidase (HRP)-conjugated secondary antibodies (Santa Cruz Biotechnology, Inc., USA) diluted in 5% PBS-skimmed milk, against the respective primary antibodies for 2 h. Protein bands were visualized by enhanced chemiluminescence (ECL) plus detection system with UV-Pro Transilluminator.

### Immunofluoroscence Assay

For the localization of TACO protein, immunofluoroscence was done on THP-1 cells by growing cells on cover slips and exposing them to PMA for 48 h at 37°C and in the presence of 5% CO_2_ to permit cell differentiation into macrophages. Two sets of cells were fixed with 4% (w/v) paraformaldehyde (PFA) in PBS for 20 min, permeabilized and blocked with 0.2% (w/v) BSA/0.1% (w/v) saponin in PBS (blocking solution) for 20 min. The cells were subsequently incubated with rabbit Coronin 1A antibody (Novus biological, USA) for 60 min at room temperature. After washing three times with PBST, cells were incubated with goat anti rabbit IgG FITC- conjugated secondary antibody (Immunology Consultants Laboratory, Inc., USA), for 60 min at 37°C. After incubation, cells were washed thrice with PBS and immediately examined by fluorescence microscopy (Olympus BX51, Japan) equipped with a UV filter system. Images were obtained with a CCD camera (Olympus, model no.E330-ADU1.2X, Japan).

### Effect of Macrophage TACO Gene Downregulation on *L. donovani* Infection *In Vitro*

Once the maximal downregulation dose of TACO mRNA was determined by treatment with the above molecules, the same dose was applied during the parasite infection study. One set of THP-1 macrophages were treated with the maximal downregulating dose of Vit.D_3_/RA and another set with CDCA/RA. After 24 h of treatment, the cells were washed two times with plain medium. Infection of macrophage was carried out using a virulent strain (DD8) of *L. donovani* grown in axenic culture. These promastigotes were added to macrophages at a ratio of 10:1. This culture was incubated for 1 h at 37°C in 5% CO_2_ incubator. Non-adherent cells were washed out and macrophages were cultivated in complete culture medium supplemented with 10% heat inactivated FBS, streptomycin (200 ug/ml), benzyl penicillin (200 U/ml) and gentamicin (40 ug/ml). Parasite load and replication rate was determined at different time intervals: early (2 and 4 h), middle (24 and 48 h) and late (72 and 96 h) stages. Infected macrophages on the cover slips were fixed and stained with Giemsa. Parasite loads were examined microscopically by counting at least 500 target macrophages for each cover slip. Efficacy of the molecules was determined by calculating the number of amastigotes per 100 macrophages at each time point.

### Statistical Analysis

Data was expressed as the mean ± SD values of triplicate samples. The statistical significance of the differences between and within groups was determined by analysis of variance (ANOVA) to define the multiple comparisons amongst different groups. The statistical analyses were performed using SPSS software. Differences were considered statistically significant for *p* < 0.05.

## Results

### Optimum Concentration of PMA for Differentiation of THP-1 Monocytes to Macrophages

**FIGURE 1:**
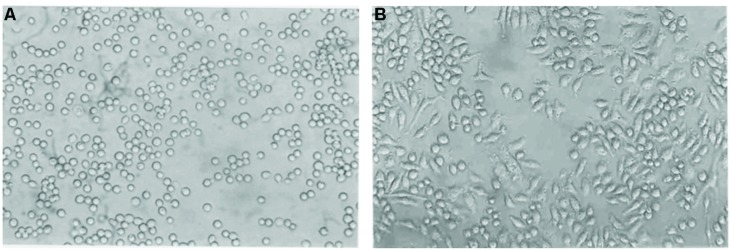
**Phase contrast micrographs of THP-1 cells in culture induced by PMA.** THP-1 cells were incubated with PMA (30 nM/ml) for 72 h. Morphological changes in THP-1 (40X) on **(A)** Day 1 **(B)** Day 3 are shown. Their morphology changed from a rounded shape to elongated and flattened.

According to the results presented in **Figure 1**, monocytes in suspension were converted to matured macrophages in 72 h after treatment with PMA. Treatment with 30 nM/ml PMA for 72 h induced changes in morphology; their rounded shape became elongated and flattened. However, attachment of cells at 20 nM/ml was unstable, and the cells were easily detached by washing. This indicated that the concentrations below 30 nM/ml PMA were not enough to differentiate the THP-1 cells stably. Hence, the minimal concentration of PMA for stable differentiation was determined to be 30 nM/ml. Cell adhesion and spreading, which are hallmarks of macrophages, were then examined under a microscope. Macrophage cells grew in a distinct morphological pattern, showing a significant raise in adhesion and spreading patterns to the growth substratum.

### Infectivity of *L. donovani* Promastigotes in Differentiated THP-1 Macrophages and TACO Gene Expression

**FIGURE 2:**
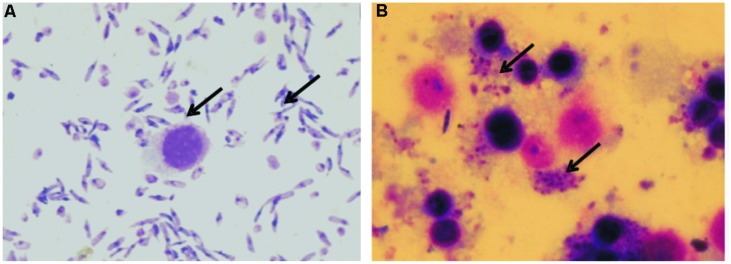
**Giemsa stained micrograph showing human macrophage cell line (THP-1) infected with *L. donovani* promastigotes. (A)** Promastigotes and Macrophages; **(B)** Macrophages showing intracellular parasite converted to amastigotes after internalization (100X; indicated by arrowheads).

Differentiated THP-1 macrophages were infected with virulent *L. donovani* promastigotes for 24 h. As demonstrated in **Figure 2**, the *L. donovani* promastigotes exhibited a marked infectivity to macrophages 24 h after infection. The uptake of parasites by mammalian cells was determined by Giemsa staining.

**FIGURE 3:**
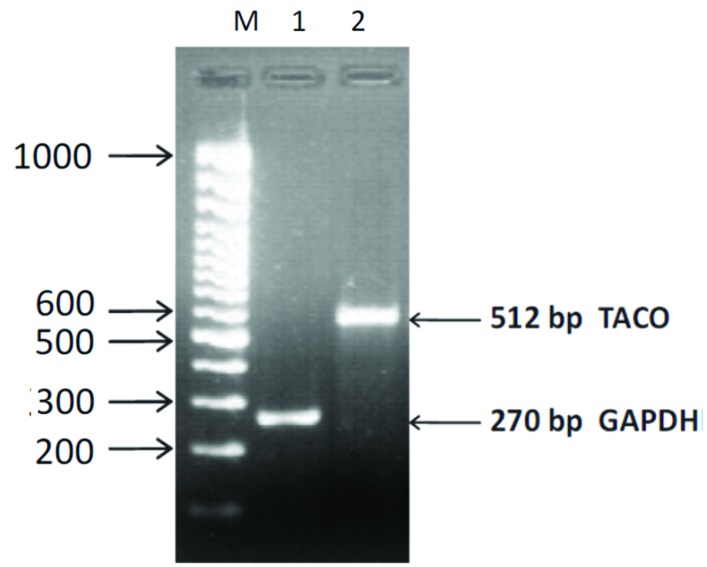
**Agarose gel photograph showing ethidium bromide stained bands of RT-PCR products of TACO and GAPDH genes.** Lane M: 100 bp DNA ladder. Lane 1: GAPDH gene; Lane2: TACO gene.

In order to determine the expression of TACO gene expression in *Leishmania* infected macrophages, RT-PCR was performed using specific primers with equal concentration of cDNAs. The house-keeping gene GADPH was amplified and used as a loading control. Gels containing RT-PCR products were photographed by UV-Pro Transilluminator camera and bands were analyzed (**Figure 3**). This assay confirmed the 512 bp TACO gene expression, when the PCR product showing ethidium bromide-stained bands obtained after electrophoresis along with the marker (**Figure 3**) and 240 bp of GAPDH gene.

Sequencing of PCR product of TACO gene (512 bp band) from agarose gel electrophoresis and BLAST search for homologous nucleotides in the database showed that 512 bp of TACO gene expression showed the highest (100%) homology to other TACO mRNAs in many organisms including humans. This confirmed that the PCR amplified gene was the exact product of TACO/Coronin 1A. This TACO gene sequence has been deposited in GenBank under the accession number GQ2141031.

### Localization and Expression of TACO in THP-1 Macrophages

**FIGURE 4:**
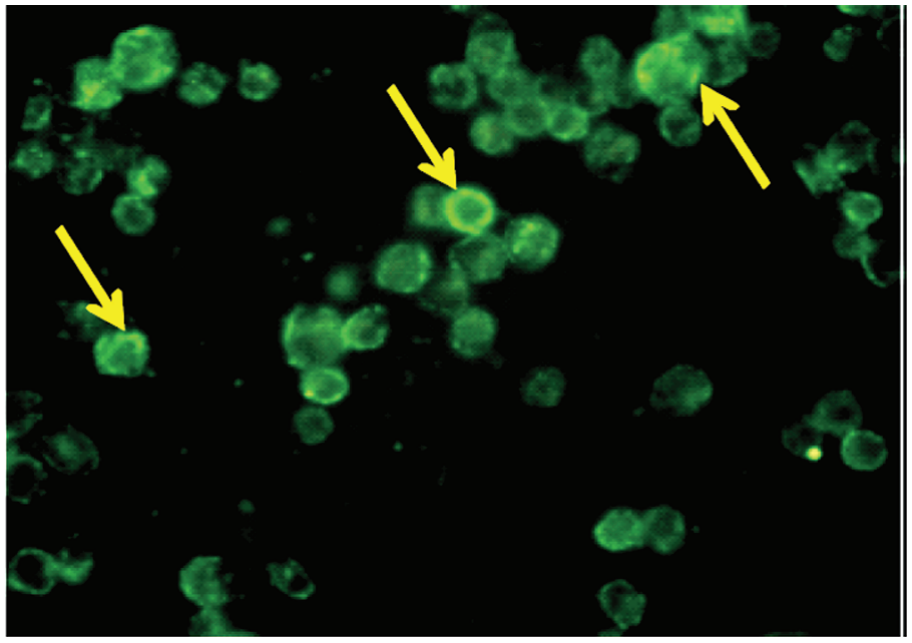
**Localization of TACO/Coronin1A in differentiated THP-1 macrophages.** Monocytes cultured on glass coverslips were differentiated into macrophages in the presence of PMA, fixed, permeabilized and stained with primary antibody, TACO/Coronin 1A Antibody, for 2 h followed by FITC labeled secondary antibody. Cells were observed under fluorescent microscope equipped with a UV filter system using 100X; indicated by arrowheads.

TACO protein localization in human THP-1 macrophages were determined by indirect immunofluoroscence using an affinity-purified antibody. Strong punctuate staining of TACO protein was observed in the cytoplasmic face of activated macrophages (**Figure 4**). Observation of this assay showed TACO protein localization on the cytoplasmic face of plasma membranes of activated THP-1 human macrophages.

**FIGURE 5:**
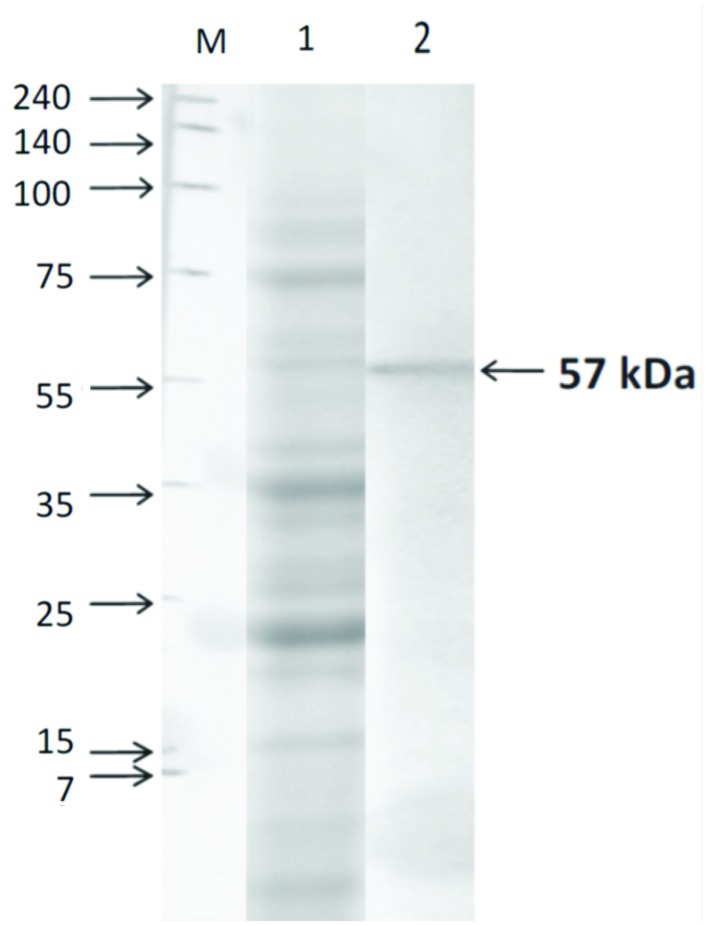
**Western blot analysis of TACO protein in THP-1 cells.** Cells were harvested after differentiation with PMA (30 nM/ml) for 72 h. Whole cell extracts were prepared and separated by SDS-PAGE on 10% gels, and Western blot assay was performed using TACO/Coronin-1A Antibody and goat anti-rabbit IgG-HRP antibody. Lane M: Marker; Lane1: SDS PAGE Profile; Lane 2: Western Blot showing the TACO protein of 57 kDa. Results are representative of at least three separate experiments that gave similar results.

Expression of TACO protein in human macrophages was confirmed by Western blot analysis using a specific antibody. Results showed that in lysates of THP-1 macrophages (**Figure 5**), anti-TACO/Coronin 1A antibody recognized a single 57 kDa protein in Western blots, confirming the expression of TACO protein.

### Vitamin D_3_/Retinoic Acid Regulates TACO Gene Expression in THP-1 Macrophages

**FIGURE 6:**
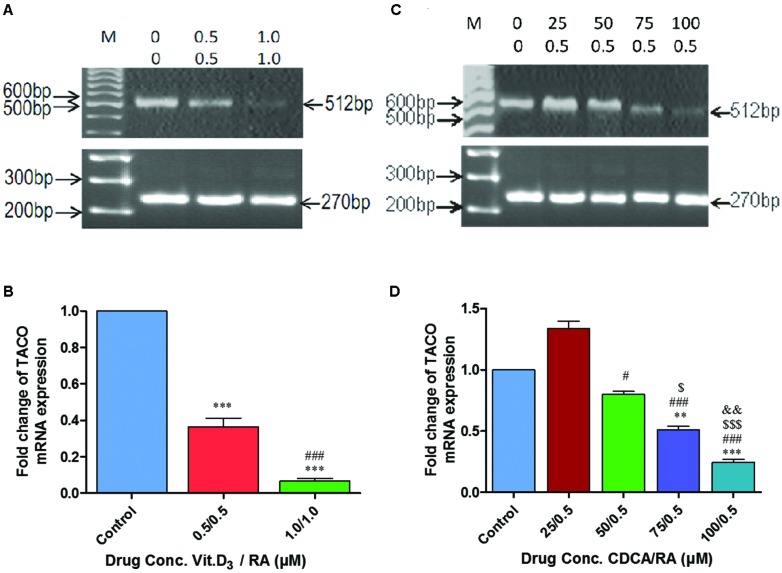
**Polymerase chain reaction (PCR) amplification showing synergistic action of Vitamin D_3_/Retinoic acid (Vit.D_3_/RA) and chenodeoxycholic acid/Retinoic acid (CDCA/RA) on mRNA expression of TACO gene. (A,C)** Representative agarose gel photographs showing ethidium bromide stained Real Time PCR products of TACO and GAPDH genes. **(B,D)** The relative levels of TACO mRNA expression represented in fold change. Each data point in the graph represents mean ± SD of three independent experiments. ***p < 0.001 (Control Vs 0.5/0.5 μM; 1.0/1.0 μM), ###*p* < 0.001 (0.5/0.5 μM Vs 1.0/1.0 μM), ***p < 0.001 (Control Vs 75/0.5 μM; 100/0.5 μM), #*p* < 0.05, ###*p* < 0.001 (25/0.5 μM Vs 50/0.5 μM; 75/0.5 μM; 100/0.5 μM), $ *p* < 0.05, $$$ *p* < 0.001 (50/0.5 μM Vs 75/0.5 μM; 100/0.5 μM), &&*p* < 0.05 (75/0.5 μM Vs 100/0.5 μM).

During this phase, to study the transcriptional regulation of TACO gene by Vit.D_3_/RA and CDCA/RA, experiments were designed to determine the optimum dose of molecules used for downregulation of TACO mRNA. Compounds from two different categories, i.e., isoprenoids derived from mevalonate pathway and vitamins (A, D), were evaluated for their effect on TACO gene expression. Exposure of THP-1 differentiated macrophages to Vit.D_3_ and RA for 24 h resulted in significant dose dependent downregulation of TACO mRNA expression. TACO mRNA level was significantly downregulated by 0.686 fold (*p* < 0.0001) in cells treated with 0.5 μM/0.5 μM Vit.D_3_/RA compared to controls. Whereas cells treated with a dose of 1.0 μM/1.0 μM Vit.D_3_/RA, mRNA level was highly downregulated by 0.949 fold (*p* < 0.0001) when compared to control groups. The effective dose on TACO mRNA downregulation of this combination was 1.0 μM/1.0 μM as compared with control groups (**Figures 6A,B**).

### Chenodeoxycholic Acid/Retinoic Acid Regulates TACO Gene Expression in a Dose Dependent Manner

Further, exposure of THP-1 macrophages to a combination of CDCA 0–100 μM and RA (0.5 μM) caused a further reduction in TACO mRNA expression levels (**Figure 6C**). Surprisingly, a combined dose of 25 μM CDCA 0.5 μM RA resulted in moderate upregulation by 0.33 fold (*p* = 0.132) of TACO mRNA compared to control groups (**Figure 6D**). In additional doses, TACO mRNA expression was downregulated at the doses of 50 μM/0.5 μM by 0.165 fold (*p* = 0.498), 75 μM/0.5 μM by 0.441 fold (*p* < 0.002) and 100 μM/0.5 μM by 0.717 fold (*p* < 0.0001) as compared to control cells. In this combination of molecules (CDCA/RA) 100 μM/0.5 μM dose was most effective in downregulation of TACO gene as compared to control groups (**Figure 6D**).

### Vitamin D_3_/Retinoic Acid and Chenodeoxycholic Acid/Retinoic Acid Inhibit Entry and Survival of *Leishmania promastigotes* in a Time Dependent Manner

**FIGURE 7:**
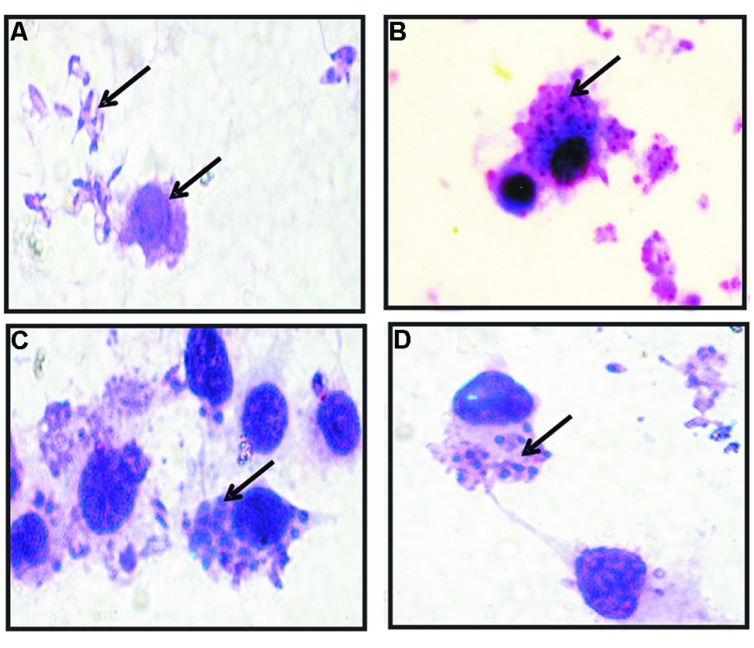
**Giemsa Stained micro-photographs of Vit.D_3_/RA and CDCA/RA pre-treated THP-1 macrophage infected with promastigotes. (A)** Promastigotes and Macrophages; **(B–D)** Conversion to amastigotes after internalization (indicated by arrowheads).

To examine the entry and intracellular survival of *L. donovani* in TACO downregulated macrophages, cells were pre-treated with maximum TACO mRNA downregulated doses of Vit.D_3_/RA and CDCA/RA (treatment before parasite infection) for 24 h. Further, to investigate the parasite survival when TACO gene is downregulated, macrophages were infected with promastigotes at 1:10 ratio (**Figure 7**), and subsequently time kinetics of THP-1 cell infection by *L. donovani* were assessed at early (2 and 4 h), middle (24 and 48 h), and late (72 and 96 h) stages. The rate of entry and survival was observed at different time intervals.

**FIGURE 8:**
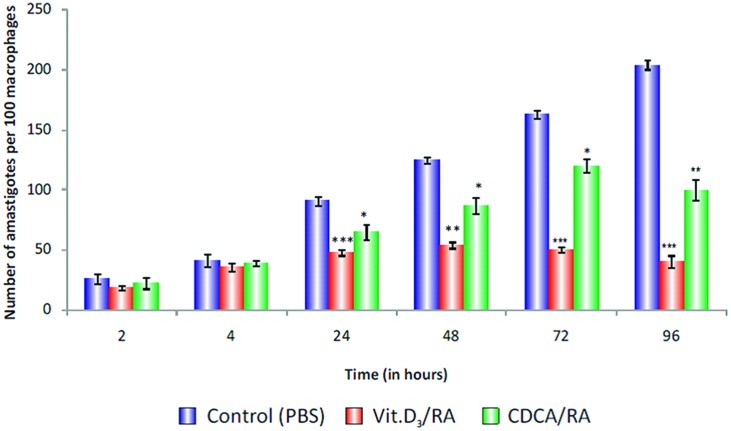
**Parasite load in Vit.D_3_/RA and CDCA/RA pre-treated THP-1 macrophage cells infected with promastigotes.** THP-1 human macrophage cell lines were pre-treated with Vit.D_3_/RA, CDCA/RA for 24 h to downregulate the TACO gene, then infected with *L. donovani* promastigotes. Parasite loads were determined by calculating the total number of amastigotes per 100 macrophages at different time intervals (2, 4, 24, 48, 72, and 96 h) post-infection. The values listed are mean ± SD of three independent cultures. Significant differences are indicated by asterisks: *p < 0.05, **p < 0.01, ***p < 0.001.

Treatment with Vit.D_3_/RA before *Leishmania* promastigote infection revealed that the entry and survival of parasites within these cells varied at different time intervals. At 2 h, 31% (*p* = 0.506) reduction and at 4 h, 13% (*p* = 0.387) reduction in parasite load was observed, but there was no significant difference between control and treated cells. At 24 h, we observed 47% (*p* < 0.0001) reduction, at 48 h 57% (*p* < 0.002) reduction, at 72 h 60% (*p* < 0.0001) reduction and at 96 h maximum reduction, i.e., 80% reduction in parasite load (*p* < 0.001; **Figure 8**) compared to the control.

Similarly, in cells after CDCA/RA pre-treatment, there was no significant difference in parasite load at 2 h (15% reduction; *p* = 0.517), and at 4 h it showed 5% reduced parasite load (*p* = 0.553). However, significant reduction in parasite loads at 24 (28%, *p* < 0.04), 48 (30%, *p* < 0.016), 72 (26%, *p* < 0.014), and 96 h (50%, *p* < 0.004) were observed compared to controls (**Figure 8**). The results showed that Vit.D_3_/RA displayed a maximum 80% (*p* < 0.001) suppression of parasite multiplication and entry of promastigote in THP-1 macrophages, while CDCA/RA treatment showed 50% reduction *(p* < 0.004; **Figure 8**).

## Discussion

Intracellular infection by the *Leishmania* parasite depends on a sequence of events including attachment, internalization, amastigote differentiation and intracellular survival. Understanding the impact of intracellular pathogens on the behavior of their host cell is the key to designing new interventions. The present study was based on the hypothesis that the transcriptional manipulation of the TACO gene leads to phagosome (carrying parasite) maturation, which will affect the survival and replication of intracellular pathogens.

In the present study, Vit.D_3_ and RA combination showed significant TACO mRNA downregulation in a dose dependent manner (**Figure 6**). Biological effects of Vit.D_3_ are mediated by vitamin D receptor (VDR), a member of a superfamily of nuclear hormone receptors (Carlberg, 2003). Similarly RA modulates transcription through the ligand dependent transcription factor RXR, which binds to a particular response element in the promoter region of target genes (Giguere, 1999). The heterodimeric complex VDR/RXR then binds to a different set of response elements within effector genes. RXR ligands have been reported to synergize with Vit.D_3_ to activate the 25 hydroxy-vitamin D_3_-24-hydroxylase promoter (Zou et al., 1997), whereas VDR/RXR ligands that have been reported to inhibit transcriptional activity of rat osteocalcin gene (MacDonald et al., 1993; Anand and Kaul, 2003) showed similar results with these molecules. Therefore, it is reasonable to speculate that Vit.D_3_ and RA act synergistically to downregulate TACO mRNA expression (Anand and Kaul, 2003).

In the present study, mevalonate-pathway derived isoprenoids, i.e., CDCA along with RA, were also used to evaluate TACO mRNA expression in a dose dependent fashion. Initially, a moderate increase in TACO mRNA expression was observed (**Figure 6**). An increased dose combination with CDCA/RA was observed to be more potent in downregulating TACO mRNA expression, initiating a pronounced downregulatory effect on TACO gene transcription (**Figure 6**). It is pertinent to note that CDCA is a potent activator of FXR, which is ligand-dependent transcription factor (Giguere, 1999). On activation, the receptor translocates into the nucleus and regulates a number of genes, either alone or in combination with its heterodimeric partner retinoid-X-receptor (RXR; Grober et al., 1999; Edwards et al., 2002). This raises the possibility that CDCA and RA, when used in combination, show a synergistic effect by activating the ligand-dependent heterodimeric complex FXR/RXR.

In support of this, Anand and Kaul (2003) demonstrated the mechanism of transcriptional repression by Vit.D_3_/RA and CDCA/RA through a planned bioinformatic approach coupled with reporter assay technology that was used to pinpoint the promoter region of the TACO gene. This region was evaluated for the presence and functional status of response elements corresponding to VDR/RXR and FXR/RXR. It has been noted that the three compounds that showed transcriptional repression of TACO mRNA expression are potent activators/inhibitors of these transcription factors (Anand and Kaul, 2003).

Keeping in view the positive role of TACO in *Leishmania* entry and survival, it is likely that downregulation of TACO mRNA could be accompanied by inhibition of leishmanial entry and survival within the macrophage. Consequently, investigation was carried out to discover whether or not treatment with these molecules suppress TACO mRNA, and their affects on *Leishmania* entry and survival. THP-1 macrophages were pre-treated with different effector molecules followed by *Leishmania* promastigote infection. The results of entry and survival of the parasite were analyzed by Giemsa staining at different time-periods.

Vit.D_3_/RA pre-treatment revealed 80% inhibition of parasite load as compared to untreated control groups (**Figure 8**). It was observed at 96 h as analyzed in *Leishmania* infected macrophages. Previously, Vit.D_3_ and RA had been reported to restrict the intracellular growth of *M. tuberculosis* (Wilkinson et al., 2000; Anand and Kaul, 2003). Similarly, Sly et al. (2001) reported that Vit.D_3_ treatment of *M. tuberculosis* infected THP-1 cells activated PI3K. PI3K was shown to be essential for the formation of the phagosomal cup (class-1A PI3K) and its fusion with lysosomes in normal cells (Fratti et al., 2001). In our study, we also observed that Vit.D_3_/RA acts synergistically and downregulates TACO gene expression significantly (**Figure 8**). Anand and Kaul (2003) reported that the effect of Vit.D_3_/RA on TACO gene expression was not evident when either was used alone, and explained that this mode of synergetic action was due to the activation of VDR/RXR transcription factors.

Similarly, CDCA/RA pre-treatment restricted *Leishmania* entry by 50% as observed at 96 h after exposure compared to control groups (**Figure 8**). Accumulating evidence suggests that bile acids modulate various signal transduction pathways including the protein kinase A, protein kinase C, c-Jun N-terminal kinase and MAP-kinase dependent cascades (Bouscarel et al., 1999; Alpini et al., 2004; De Fabiani et al., 2004). Further, no previous study has shown a direct link between CDCA and *Leishmania.* The results of the experiments in this study indicate a potential role of CDCA in killing *Leishmania.*

**FIGURE 9:**
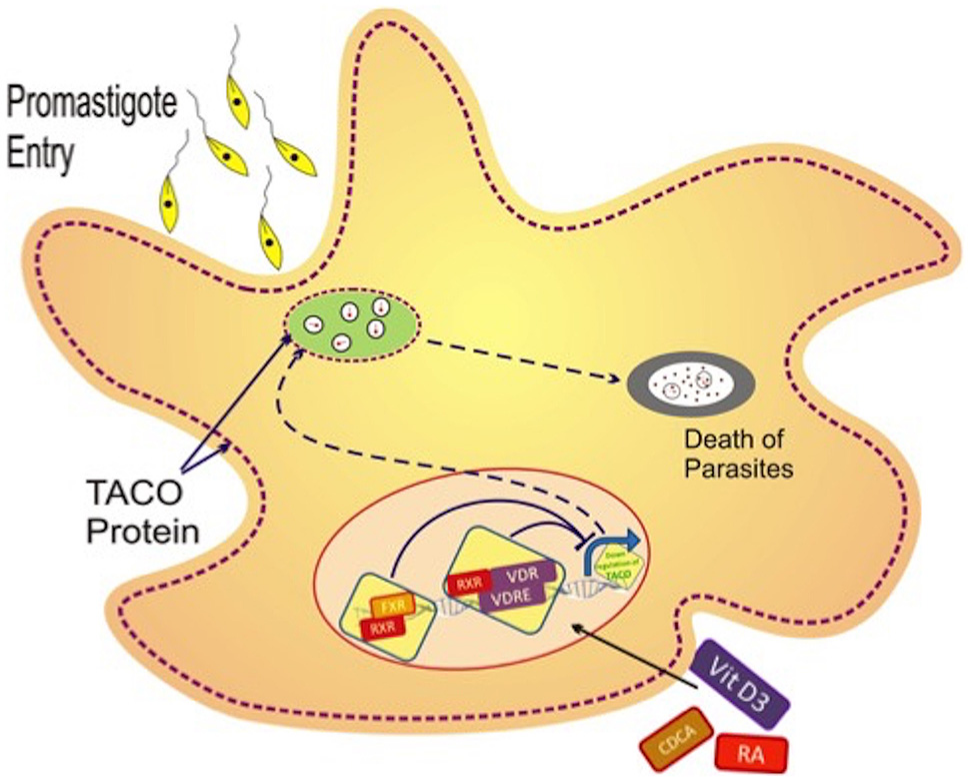
**Hypothetical model of transcriptional regulation of TACO gene with various effector molecules.** *Leishmania* promastigotes enter the macrophage by phagocytosis, after which phagosomes are formed that retain tryptophan–aspartate containing coat protein (TACO). Such retention of TACO may support survival of the parasite along with late maturation and late fusion with late endosomes. Vit.D_3_/RA and CDCA/RA treatment leads to the downregulation of the *TACO* gene, which is possibly responsible for the detachment of TACO protein over the phagosome, and consequently, early maturation and phagolysosomal killing of the parasite.

Altogether, the results reveal that Vit.D3/RA and CDCA/RA suppress TACO mRNA expression to significant levels. Such expression has been reported to operate through the activity of transcription factors VDR/RXR and FXR/RXR. Consequently, TACO gene suppression with these molecules led to inhibition of *Leishmania* entry and survival, thus proving Vit.D3/RA and CDCA/RA combinations, we found that Vit.D_3_/RA combination showed highly effective levels of parasite suppression compared with the CDCA/RA combination. These observations suggest that TACO gene downregulation may modulate the invasion of parasite phagolysosomal maturation in macrophages following phagocytosis of *L. donovani* carrying promastigotes (**Figure 9**). The current study provides evidence that TACO gene downregulation modulates parasite entry and survival in macrophages following the phagocytosis of *L. donovani* promastigotes.

The conclusions derived from this study suggest that the effect of TACO gene downregulation can help elucidate the pathophysiology of host and parasite interactions, thereby unraveling the probable potential preventive and therapeutic alternatives against leishmaniasis. To the best of our knowledge, this study is the first to report the effect of TACO gene downregulation at the transcriptional level using vitamins and isoprenoids on *L. donovani* infection, which could prove to be a basis for the development of novel approaches in the control of leishmaniasis.

## Conflict of Interest Statement

The authors declare that the research was conducted in the absence of any commercial or financial relationships that could be construed as a potential conflict of interest.
